# Factors Associated with Functional Outcome Following Acute Ischemic Stroke Due to M1 MCA/ICA Occlusion in the Extended Time Window

**DOI:** 10.3390/jcm14155556

**Published:** 2025-08-06

**Authors:** John Constantakis, Quinn Steiner, Thomas Reher, Timothy Choi, Fauzia Hollnagel, Qianqian Zhao, Nicole Bennett, Veena A. Nair, Eric E. Adelman, Vivek Prabhakaran, Beverly Aagard-Kienitz, Bolanle Famakin

**Affiliations:** 1 Berbee Walsh Department of Emergency Medicine, University of Wisconsin Hospitals and Clinics, Madison, WI 53792, USA; constantakis@wisc.edu; 2 Department of Neurology, University of Wisconsin School of Medicine and Public Health, Medical Foundation Centennial Building, 1685 Highland Ave, Madison, WI 53705, USA; qsteiner@wisc.edu (Q.S.); adelman@neurology.wisc.edu (E.E.A.); 3 Monument Health Rapid City Hospital, Rapid City, SD 57701, USA; treher@dakota-radiology.com; 4 Department of Radiology, UW Health University Hospital, 600 Highland Ave, Madison, WI 53792, USA; tjchoi@mednet.ucla.edu (T.C.); vnair@uwhealth.org (V.A.N.); vprabhakaran@uwhealth.org (V.P.); baagaard-kienitz@uwhealth.org (B.A.-K.); 5 Department of Internal Medicine, University of Wisconsin School of Medicine and Public Health, Madison, WI 53705, USA; fhollnagel@medicine.wisc.edu; 6 Department of Biostatistics and Medical Informatics, University of Wisconsin School of Medicine and Public Health, Madison, WI 53726, USA; qzhao36@wisc.edu; 7 University of Wisconsin Hospitals and Clinics, 600 Highland Ave, Madison, WI 53792, USA; nbennett@uwhealth.org

**Keywords:** extended thrombectomy time window, M1 occlusion, internal carotid occlusion, outcomes, ischemic stroke, functional outcome, predictors of functional outcome and stroke, preexisting hypertension, acute ischemic stroke

## Abstract

**Introduction:** A validated clinical decision tool predictive of favorable functional outcomes following endovascular thrombectomy (EVT) in acute ischemic stroke (AIS) remains elusive. We performed a retrospective case series of patients at our regional Comprehensive Stroke Center, over a four-year period, who have undergone EVT to elucidate patient characteristics and factors associated with a favorable functional outcome after EVT. **Methods:** We reviewed all cases of EVT at our institution between February 2018 and February 2022 in the extended time window from 6–24 h. Demographic, clinical, imaging, and procedure co-variates were included. A favorable clinical outcome was defined as a modified Rankin scale of 0–2. We included patients with M1 or internal carotid artery occlusion treated with EVT within 6–24 h after symptom onset. We used a univariate and multivariate logistic regression analysis to identify patient factors associated with a favorable clinical outcome at 90 days. **Results:** Our study included evaluation of 121 patients who underwent EVT at our comprehensive stroke center. Our analysis demonstrates that a higher recanalization score based on the modified Thrombolysis In Cerebral Infarction (mTICI) scale (2B-3) was a strong indicator of a favorable outcome (OR 7.33; CI 2.06–26.07; *p* = 0.0021). Our data also showed that a higher baseline National Institutes of Health Stroke Scale (NIHSS) score (*p* = 0.0095) and the presence of pre-existing hypertension (*p* = 0.0035) may also be predictors of an unfavorable outcome (mRS > 2) per our multivariate analysis. Conclusion: Patients without pre-existing hypertension had more favorable outcomes following EVT in the expanded time window. This is consistent with other multicenter data in the expanded time window that demonstrates greater odds of a poor outcome with elevated pre-, peri-, and post-endovascular-treatment blood pressure. Our data also demonstrate that the mTICI score is a strong predictor of favorable outcome, even after controlling for other variables. A lower baseline NIHSS at the time of thrombectomy may also indicate a favorable outcome. Furthermore, the presence of clinical or radiographic mismatch based on the Alberta Stroke Program Early Computed Tomography Score (ASPECTS) and NIHSS per DAWN and DEFUSE-3 criteria did not emerge as a predictor of favorable outcome, which is congruent with recent randomized controlled trials and meta-analyses.

## 1. Introduction

Ischemic stroke incidence, prevalence, and mortality are increasing annually, with this trend expected to continue [1]. Endovascular intervention for stroke has emerged as an effective treatment strategy in patients with an acute ischemic stroke secondary to large vessel occlusion (LVO) [2]. Initially, significant clinical benefit was seen when endovascular intervention was performed within 6 h after the onset of stroke symptoms [3,4]. However, the recent DEFUSE-3 and DAWN trials expanded this time window out to 6–24 h [5,6].

Although the guidelines for the acute treatment of stroke have evolved to include this expanded time window from 6–24 h, even according to our study, there are large variations in patient outcomes. Within this expanded time window, 40–50% of patients have poor neurological outcomes per modified Rankin scores (mRS) (defined as mRS 3-6), with mortality rates around 15% [5,6,7,8]. A variety of factors have been identified as correlating with worse clinical outcomes following stroke [9,10]; however, a clinically usable model for outcome prognostication has remained elusive.

We sought to examine our single-center experience at a major, regional, comprehensive stroke center with endovascular intervention for acute ischemic stroke in patients with M1 middle cerebral artery (MCA) and internal carotid artery (ICA) occlusions. Patients with these types of LVOs typically have a worse prognosis [5,6]. These LVOs constitute many patients that undergo endovascular intervention [5,6]. Consequently, understanding factors that impact outcomes may greatly improve quality of care.

The purpose of our investigation is to identify potential clinical and imaging biomarkers predictive of a favorable functional outcome following acute stroke secondary to M1 and ICA occlusion during the extended time window. This would ideally be used to predict pre-procedurally which patients would show the greatest functional outcome response to thrombectomy. We looked at multiple clinical and imaging variables such as hypertension, atrial fibrillation, ejection fraction, ASPECTS, and others in an effort to identify both positive and negative variables associated with favorable functional outcomes.

## 2. Methods

### 2.1. Patient Selection

After approval from our institutional review board, we retrospectively reviewed all patients admitted or transferred to our hospital who underwent endovascular stroke treatment for arterial ischemic stroke from 6–24 h after symptom onset between February 2018 and February 2022. The decision to perform the endovascular procedure was made on an individual basis with consensus between the neuro-interventionalist and neurology team. We included patients older than 18 years who underwent endovascular intervention for occlusion of M1 vessels as well as either intracranial or cervical internal carotid artery (ICA) vessels, had baseline imaging (which included non-contrast CT head, CT angiography head and neck, and CT perfusion) performed at the University of Wisconsin University Hospital, and presented with symptom onset of greater than 6 but less than 24 h.

All procedures were performed according to standard-of-care guidelines [5,6]. We collected baseline clinical and radiological characteristics, procedure details, and outcomes, including the degree of successful recanalization according to the modified Thrombolysis In Cerebral Infarction (mTICI) scale (grade of 0–3, with higher scores indicating greater recanalization). Specifically, a grade is assigned at the end of each procedure, with mTICI ≤ 2b indicating incomplete recanalization, and mTICI 2c or 3 indicating near-complete or complete recanalization, respectively. Comorbid variables including atrial fibrillation, diabetes, hypertension, hyperlipidemia, coronary artery disease, peripheral artery disease, and ejection fraction were also analyzed. The presence of comorbidities was assessed using the presence of ICD-10 codes as presented in the patients’ chart via electronic medical record chart review.

We defined a favorable outcome as a modified Rankin score (mRS) of 0–2 at 90 days post-presentation. This was calculated by chart review of physical therapy follow-up notes at 90 days and verified between two operators certified in mRS scoring via WebDCU and Neurological Emergencies Treatment Trials Network.

We also evaluated whether each patient taken for thrombectomy met DAWN and DEFUSE-3 trial criteria based on clinical or radiographic mismatch and if meeting these criteria was associated with favorable outcome. Specifically, patients were classified as meeting either DAWN [6] criteria such as less than one third MCA territory involved as evidenced by CT or MRI, occlusion of intracranial ICA and/or MCA M1 as evidenced by CTA or MRA, and clinical imaging mismatch as identified on CT perfusion or MR DWI defined as 0–20 cc core infarct and NIHSS > 10 (and age >80 years old), 0–30 cc core infarct and NIHSS > 10 (and age less than 80 years old), or 31 cc to <50 cc infarct and NIHSS > 20 (and age <80 years old), or DEFUSE-3 Criteria [5] including ischemic core <70 cc, a ratio of volume of ischemic tissue to initial infarct volume of 1.8 or more, and an absolute volume of potentially reversible ischemia of 15 mL or more.

### 2.2. Statistical Methodology

#### Data Analyses

Descriptive statistics were calculated for all variables, including counts and frequencies for categorical variables, and means with standard deviations or medians with interquartile ranges for continuous variables. Comparisons between favorable outcome groups were conducted using chi-square or Fisher’s exact tests for categorical variables, and Student’s *t*-test or the non-parametric Wilcoxon rank-sum test for continuous variables. To identify predictors of favorable outcomes, baseline characteristics and medical history variables with *p*-value = 0.20 in the univariate group comparisons were included as candidate predictors in the multivariate logistics regression model. Stepwise and forward selection methods yielded the same final model, for which odds ratios and 95% confidence intervals were reported. Differences in outcomes were graphically assessed using coefficient plots with extended confidence intervals. A complete case analysis was employed, and variables with more than 50% missing data were excluded from the regression. Mortality and discharge disposition were not analyzed due to quasi-complete separation with the outcomes. Patients were classified as expired if death occurred between discharge and 90-day follow-up phone call versus in-hospital mortality if they died between procedure and discharge. Statistical significance was defined as a *p*-value < 0.05. All statistical analyses were conducted using SAS Version 9.4 (Cary, NC, USA) and STATA version 17.

## 3. Results

**Figure 1:**
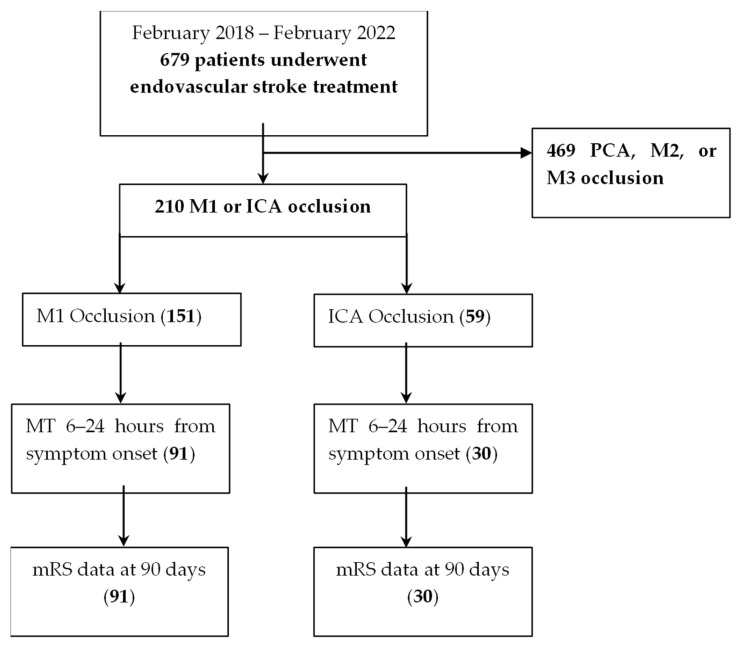
Patient flowchart.

A total of 679 patients underwent thrombectomy at our comprehensive stroke center over a four-year period. Of these, 121 (18%) met inclusion criteria (CT or MR perfusion on arrival, carotid, or M1 occlusion) (Figure 1) and also had satisfactory data complements, meaning that the variable contained > 50% of the investigated data fields.

### 3.1. Patient Cohort Characteristics and Outcomes

Baseline characteristics are summarized in Table 1. There were no significant differences between groups in terms of occlusion site, sex, or smoking history, or diagnosis of diabetes, atrial fibrillation, decreased cardiac ejection fraction, or cervical carotid involvement. Favorable clinical outcomes were observed in 51 (42%) patients, including 9 (18%) with ICA occlusions and 42 (82%) with M1 occlusions. Favorable clinical outcomes were associated with younger age, lower baseline NIHSS scores, shorter hospital length of stay, higher mTICI scores, longer symptom onset-to-groin puncture time, and absence of pre-existing hypertension. Favorable outcomes also mean less in-hospital mortality and better discharge disposition.

### 3.2. Predictors of Favorable Outcomes

Logistic regression analysis was used to evaluate factors associated with favorable outcomes in the studied cohort. While age, length of stay, and longer symptom onset-to-groin puncture time were associated with favorable outcomes in univariate analyses, they did not remain significant predictors in the multivariable model. They may not independently predict favorable outcomes once other variables are considered.

Ejection fraction did not demonstrate a significant association with favorable outcomes in either univariate or multivariate analyses, suggesting that it may not be a strong predictor of favorable outcomes in this cohort. Interestingly, whether or not patients met DAWN or DEFUSE-3 criteria [5,6] also did not significantly predict a favorable outcome (*p*-value 0.913 and 0.337, respectively).

**Figure 2:**
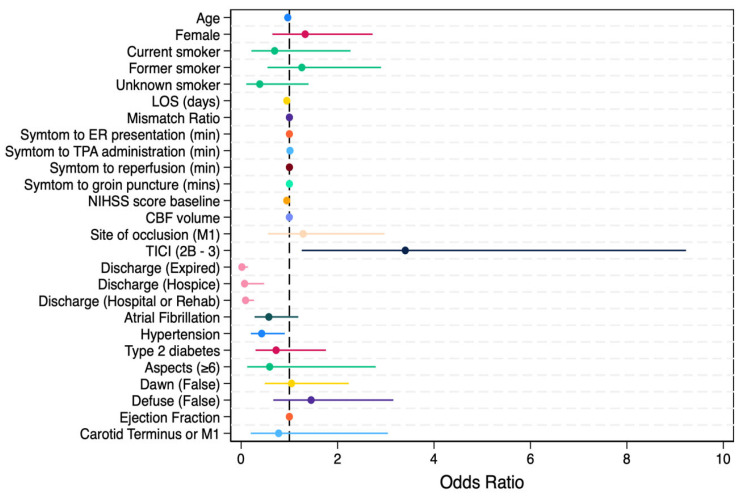
Forest plot of odds ratio of variables and their association with thrombectomy outcome.

Notably, the absence of pre-existing hypertension, lower baseline NIHSS scores, and achieving higher levels of reperfusion (as indicated by a mTICI score of 2B-3) emerged as strong predictors of favorable outcomes maintaining their significance in both univariate and multivariate analysis (Table 2). These data are reflected in a forest plot of the variable odds ratios and their associations with thrombectomy outcomes (Figure 2).

## 4. Discussion

This single-center, retrospective study aimed to identify clinical and imaging predictors of favorable outcomes following endovascular stroke treatment in patients presenting with proximal ICA or M1 MCA occlusions in the extended time window. We acknowledge several limitations of our study, including the retrospective, single-center design and our relatively small sample size, which may have limited statistical power for some analyses. We also recognize that several variables (mainly ‘symptom onset to groin puncture between 9–12 h in the favorable mRS group’) had zero observations, which could limit statistical reliability in some odds ratios. Notable findings included the role of a pre-existing diagnosis of hypertension as being associated with an unfavorable functional outcome.

Our data also reflected no significant difference in functional outcomes regardless of whether DAWN (*p* value 0.913) or DEFUSE 3 (*p* value 0.337) clinical mismatch criteria were chosen. This is in line with several recent RCTs and meta-analyses demonstrating improved clinical outcomes with mechanical thrombectomy regardless of which clinical mismatch criteria were chosen.

The AURORA study [11], a pooled analysis of six RCTs including DAWN and DEFUSE-3, sought to determine whether selecting patients based on clinical mismatch (DAWN criteria) offers any advantage over selection based on perfusion mismatch (DEFUSE-3 criteria). Importantly, it found a similar benefit in terms of functional independence (mRS 0–2) at 90 days regardless of which mismatch criteria were chosen. This finding was reflected in our dataset that showed no statistical impact on outcome whether DAWN or DEFUSE-3 criteria were used.

Further research is investigating what degree of mismatch is necessary for functional benefit to be achieved, but recent studies are demonstrating that benefit is possible beyond the mismatch criteria defined in the DAWN and DEFUSE-3 studies. Recently published RCTs such as SELECT-2 [12] and RESCUE-JAPAN LIMIT [13] have shown that patients with a low ASPECTS (which is generally considered to correlate to the area of core infarct) have greater odds of achieving functional independence with mechanical thrombectomy compared with undergoing medical therapy alone. Both RCTs along with other observational studies and meta-analyses have already demonstrated the benefit of endovascular stroke treatment despite a low ASPECTS (defined as ASPECTS 2–5) [7,14]. This is consistent with our data that showed no statistically significant association with ASPECTS and a favorable endovascular stroke treatment outcome. Further, this also suggests that the degree of recanalization, as demonstrated by the post-procedural mTICI score, is also the more critical indicator of prognosis, which has also been shown in the literature [15].

A notable finding in our study was the significant association between pre-existing hypertension and unfavorable outcomes (mRS > 2) (OR 0.33; *p*-value 0.0135; 95% CI 0.14–0.80). This aligns with other multicenter data showing that elevated blood pressure before, during, and after endovascular treatment is linked to poorer outcomes [16]. Possible explanations identified include an increased risk of symptomatic intracerebral hemorrhage (sICH) and reduced rates of successful recanalization.

Our data also demonstrated that the presence of symptomatic post-endovascular treatment ICH was more likely to be associated with a less favorable outcome and higher post-procedure mRS. This is consistent with other investigations that have demonstrated an association between hypertension and post-endovascular treatment sICH. However, the causality association was not investigated in our data, and whether patients with pre-existing hypertension are more likely to have sICH is still an open question. This is an area of interest to be examined in future analyses.

The greater driving factor of this negative association may be more related to decreased odds of successful recanalization. A strong association between hypertension and increased vessel tortuosity is well established and could result in reduced ability to pass an angio-catheter to the site of occlusion and overall success of endovascular stroke treatment [17]. Another interesting hypothesis suggests that uncontrolled hypertension may be inversely associated with the development of collateral flow. This would result in a greater hemodynamic force imparted on the area of occlusion and could potentially cause clot impaction and impaired ability for mechanical clot retrieval [18].

As expected, our data confirmed that higher mTICI scores (*p*-value 0.0021) and lower baseline NIHSS scores on presentation (*p*-value 0.0095) were associated with favorable outcomes. Negative associations demonstrated hypertension more likely to be associated with a poor functional outcome (mRS > 0–2, *p*-value 0.0135). Overall, these data contribute to the growing body of literature aimed at refining patient selection criteria for endovascular stroke treatment in the expanded thrombectomy time window.

## Figures and Tables

**Table 1:** Baseline characteristics and medical history of cohort.

Variables	Overall	Favorable	Unfavorable	*p*
	MRS 0–6	MRS 0–2	MRS 3–6	
	N = 121	N = 51	N = 70	
Age, Mean (SD)	73.4 (14.7)	68.7 (14.6)	76.8 (13.9)	0.002 *
Age > = 80				
No	72 (59.5%)	37 (72.6%)	35 (50.0%)	0.013 *
Yes	49 (40.5%)	14 (27.5%)	35 (50.0%)	
Sex (Female)				
Female	58 (47.9%)	26 (51.0%)	32 (45.7%)	0.567
Male	63 (52.1%)	25 (49.0%)	38 (54.3%)	
Smoking				
Current smoker	16 (13.2%)	6 (11.8%)	10 (14.3%)	0.463
Former smoker	48 (39.7%)	22 (43.1%)	26 (37.1%)	
Never smoker	41 (33.9%)	19 (37.3%)	22 (31.4%)	
N/A	16 (13.2%)	4 (7.8%)	12 (17.1%)	
LOS, Median (IQR)	6 (3.14–11)	4.12 (3–7)	7.20 (4.24–14)	<0.001 *
Mismatch volume, mean (SD)	95.467 (49.218)	93.818 (47.309)	96.619 (50.852)	0.774
Mismatch volume ≥ 40				
No	13 (10.7%)	6 (11.8%)	7 (10.0%)	0.757
Yes	108 (89.3%)	45 (88.2%)	63 (90.0%)	
Symptom onset to er presentation (hour)				0.329
Mean (SD)	10.32 (4.51)	10.81 (4.83)	9.96 (4.26)	
Median (IQR)	9.92 (6.32–13.06)	10.37 (6.82–13.75)	9.78 (6.17–12.75)	
Symptom onset to reperfusion (hour)				0.141
Mean (SD)	12.60 (4.30)	13.28 (4.56)	12.05 (4.04)	
Median (IQR)	12.23 (8.77–15.34)	13.52 (8.92–16.28)	11.66 (8.60–14.39)	
Symptom onset to groin puncture (hour)				0.037 *
Mean (SD)	11.50 (4.32)	12.38 (4.43)	10.82 (4.14)	
Median (IQR)	11.23 (7.43–14.12)	12.08 (8.05–15.50)	10.01 (6.92–13.03)	
Symptom onset to groin puncture				
6–<9 h	41 (35.0%)	15 (29.4%)	26 (39.4%)	0.209
9–<12 h	2 (1.7%)	0 (0.0%)	2 (3.0%)	
12+ h	74 (63.2%)	36 (70.6%)	38 (57.6%)	
Baseline NIHSS, Median (IQR)	13.5 (7–20)	12 (6–17)	15 (9–21)	0.014 *
Site of Occlusion				
ICA	30 (24.8%)	9 (17.7%)	21 (30.0%)	0.120
M1	91 (75.2%)	42 (82.4%)	49 (70.0%)	
CBF (cc), Mean (SD)	17.240 (26.126)	15.796 (24.053)	18.30 (27.70)	0.631
CBF				
<10 cc	62 (51.7%)	27 (54.0%)	35 (50.0%)	0.787
10–25 cc	13 (10.8%)	6 (12.0%)	7 (10.0%)	
>25 cc	45 (37.5%)	17 (34.0%)	28 (40.0%)	
TICI				
0–2A	26 (21.5%)	4 (7.8%)	22 (31.4%)	0.002 *
2B–3	95 (78.5%)	47 (92.2%)	48 (68.6%)	
Discharge disposition				
Expired	13 (10.7%)	0 (0%)	13 (18.6%)	<0.001 *
Home	33 (27.3%)	28 (54.9%)	5 (7.1%)	
Hospice	7 (5.8%)	0 (0%)	7 (10.0%)	
Hospital or Rehab	68 (56.2%)	23 (45.1%)	45 (64.3%)	
In-Hospital Mortality	10 (8.3%)	0 (0%)	10 (14.3%)	0.005 *
Atrial fibrillation				
No	58 (47.9%)	29 (56.9%)	29 (41.4%)	0.093
Yes	63 (52.1%)	22 (43.1%)	41 (58.6%)	
Hypertension				
No	45 (37.2%)	26 (51.0%)	19 (27.1%)	0.007 *
Yes	76 (62.8%)	25 (49.0%)	51 (72.9%)	
Type-2 diabetes				
No	95 (78.5%)	42 (82.4%)	53 (75.7%)	0.380
Yes	26 (21.5%)	9 (17.7%)	17 (24.3%)	
ASPECTS				
2–5	7 (7.0%)	4 (9.5%)	3 (5.2%)	0.449
≥6	93 (93.0%)	38 (90.5%)	55 (94.8%)	
Dawn [6]				
True	41 (33.9%)	17 (33.3%)	24 (34.3%)	0.913
False	80 (66.1%)	34 (66.7%)	46 (65.7%)	
Defuse [5]				
True	39 (32.2%)	14 (27.5%)	25 (35.7%)	0.337
False	82 (67.8%)	37 (72.6%)	45 (64.3%)	
Symptomatic ICH				
No	116 (95.9%)	51 (100.0%)	65 (92.9%)	0.073
Yes	5 (4.1%)	0 (0.0%)	5 (7.1%)	
EF	58.972 (13.834)	58.404 (12.942)	59.417 (14.587)	0.709
TPA given				
No	44 (83.0%)	21 (87.5%)	23 (79.3%)	0.488
Yes	9 (17.0%)	3 (12.5%)	6 (20.7%)	
Symptom onset to TPA administration (hour)				0.154
Mean (SD)	3.16 (1.66)	4.40 (2.48)	2.85 (1.37Z)	
Median (IQR)	3.50 (2.00–4.15)	3.67 (2.37–7.17)	3.25 (1.50–4.08)	
Cervical Carotid involvement				
No	10 (17.2%)	5 (20.0%)	5 (15.2%)	0.732
Yes	48 (82.8%)	20 (80.0%)	28 (84.9%)	

This table presents baseline characteristics and medical history of a cohort, categorized by favorable (MRS 0–2) and unfavorable (MRS 3–6) outcomes, with an overall grouping of MRS 0–6. * Statistically significant at *p* ≤ 0.05. Abbreviations: LOS = Length of Stay, ER = Emergency Room, TPA = Tissue Plasminogen Activator, SD = standard deviation, mRS = modified Rankin Score, IQR = interquartile range, NIHSS = national institute of health stroke scale, ICA = internal carotid artery, MCA = middle cerebral artery, CBF = Cerebral Blood Flow (i.e., volume of ischemic tissue), mTICI = Modified Thrombolysis in Cerebral Infarction, ASPECTS = Alberta stroke program early computed tomography score, DEFUSE = DEFUSE 3 Trial, DAWN = DAWN Trial.

**Table 2:** Logistic regression table presents predictors of a favorable outcome (MRS 0–2).

Variables	OR	95% CI	P
Baseline NIHSS	0.92	0.87–0.98	0.0095
mTICI, 2B–3 vs. 0–2A	7.33	2.06–26.07	0.0021
Hypertension, Yes vs. No	0.33	0.14–0.80	0.0135

## Data Availability

The data presented in this study are available on request from the corresponding author due to HIPPAA and privacy reasons.
